# Solving the Credit Assignment Problem With the Prefrontal Cortex

**DOI:** 10.3389/fnins.2018.00182

**Published:** 2018-03-27

**Authors:** Alexandra Stolyarova

**Affiliations:** Department of Psychology, University of California, Los Angeles, Los Angeles, CA, United States

**Keywords:** orbitofrontal, dorsolateral prefrontal, anterior cingulate, learning, reward, reinforcement, plasticity, behavioral flexibility

## Abstract

In naturalistic multi-cue and multi-step learning tasks, where outcomes of behavior are delayed in time, discovering which choices are responsible for rewards can present a challenge, known as the *credit assignment problem*. In this review, I summarize recent work that highlighted a critical role for the prefrontal cortex (PFC) in assigning credit where it is due in tasks where only a few of the multitude of cues or choices are relevant to the final outcome of behavior. Collectively, these investigations have provided compelling support for specialized roles of the orbitofrontal (OFC), anterior cingulate (ACC), and dorsolateral prefrontal (dlPFC) cortices in contingent learning. However, recent work has similarly revealed shared contributions and emphasized rich and heterogeneous response properties of neurons in these brain regions. Such functional overlap is not surprising given the complexity of reciprocal projections spanning the PFC. In the concluding section, I overview the evidence suggesting that the OFC, ACC and dlPFC communicate extensively, sharing the information about presented options, executed decisions and received rewards, which enables them to assign credit for outcomes to choices on which they are contingent. This account suggests that lesion or inactivation/inhibition experiments targeting a localized PFC subregion will be insufficient to gain a fine-grained understanding of credit assignment during learning and instead poses refined questions for future research, shifting the focus from focal manipulations to experimental techniques targeting cortico-cortical projections.

## Introduction

When an animal is introduced to an unfamiliar environment, it will explore the surroundings randomly until an unexpected reward is encountered. Reinforced by this experience, the animal will gradually learn to repeat those actions that produced the desired outcome. The work conducted in the past several decades has contributed a detailed understanding of the psychological and neural mechanisms that support such reinforcement-driven learning (Schultz and Dickinson, 2000; Schultz, 2004; Niv, 2009). It is now broadly accepted that dopamine (DA) signaling conveys prediction errors, or the degree of surprise brought about by unexpected rewards, and interacts with cortical and basal ganglia circuits to selectively reinforce the advantageous choices (Schultz, 1998a,b; Schultz and Dickinson, 2000; Niv, 2009). Yet, in naturalistic settings, where rewards are delayed in time, and where multiple cues are encountered, or where several decisions are made before the outcomes of behavior are revealed, discovering which choices are responsible for rewards can present a challenge, known as the *credit assignment problem* (Mackintosh, 1975; Rothkopf and Ballard, 2010).

In most everyday situations, the rewards are not immediate consequences of behavior, but instead appear after substantial delays. To influence future choices, the teaching signal conveyed by DA release needs to reinforce synaptic events occurring on a millisecond timescale, frequently seconds before the outcomes of decisions are revealed (Izhikevich, 2007; Fisher et al., 2017). This apparent difficulty in linking preceding behaviors caused by transient neuronal activity to a delayed feedback has been termed the *distal reward* or *temporal* credit assignment problem (Hull, 1943; Barto et al., 1983; Sutton and Barto, 1998; Dayan and Abbott, 2001; Wörgötter and Porr, 2005). Credit for the reward delayed by several seconds can frequently be assigned by establishing an eligibility trace, a molecular memory of the recent neuronal activity, allowing modification of synaptic connections that participated in the behavior (Pan et al., 2005; Fisher et al., 2017). On longer timescales, or when multiple actions need to be performed sequentially to reach a final goal, intermediate steps themselves can acquire motivational significance and subsequently reinforce preceding decisions, such as in temporal-difference (TD) learning models (Sutton and Barto, 1998).

Several excellent reviews have summarized the accumulated knowledge on mechanisms that link choices and their outcomes through time, highlighting the advantages of eligibility traces and TD models (Wörgötter and Porr, 2005; Barto, 2007; Niv, 2009; Walsh and Anderson, 2014). Yet these solutions to the distal reward problem can impede learning in multi-choice tasks, or when an animal is presented with many irrelevant stimuli prior to or during the delay. Here, I only briefly overview the work on the distal reward problem to highlight potential complications that can arise in credit assignment based on eligibility traces when learning in multi-cue environments. Instead, I focus on the *structural* (or *spatial*) credit assignment problem, requiring animals to select and learn about the most meaningful features in the environment and ignore irrelevant distractors. Collectively, the reviewed evidence highlights a critical role for the prefrontal cortex (PFC) in such contingent learning.

Recent studies have provided compelling support for specialized functions of the orbitofrontal (OFC) and dorsolateral prefrontal (dlPFC) cortices in credit assignment in multi-cue tasks, with fewer experiments targeting the anterior cingulate cortex (ACC). For example, it has seen suggested that the dlPFC aids reinforcement-driven learning by directing attention to task-relevant cues (Niv et al., 2015), the OFC assigns credit for rewards based on the causal relationship between trial outcomes and choices (Jocham et al., 2016; Noonan et al., 2017), whereas the ACC contributes to unlearning of action-outcome associations when the rewards are available for free (Jackson et al., 2016). However, this work has similarly revealed shared contributions and emphasized rich and heterogeneous response properties of neurons in the PFC, with different subregions monitoring and integrating the information about the task (i.e., current context, available options, anticipated rewards, as well as delay and effort costs) at variable times within a trial (upon stimulus presentation, action selection, outcome anticipation, and feedback monitoring; ex., Hunt et al., 2015; Khamassi et al., 2015). In the concluding section, I overview the evidence suggesting that contingent learning in multi-cue environments relies on dynamic cortico-cortical interactions during decision making and outcome valuation.

## Solving the temporal credit assignment problem

**Figure 1:**
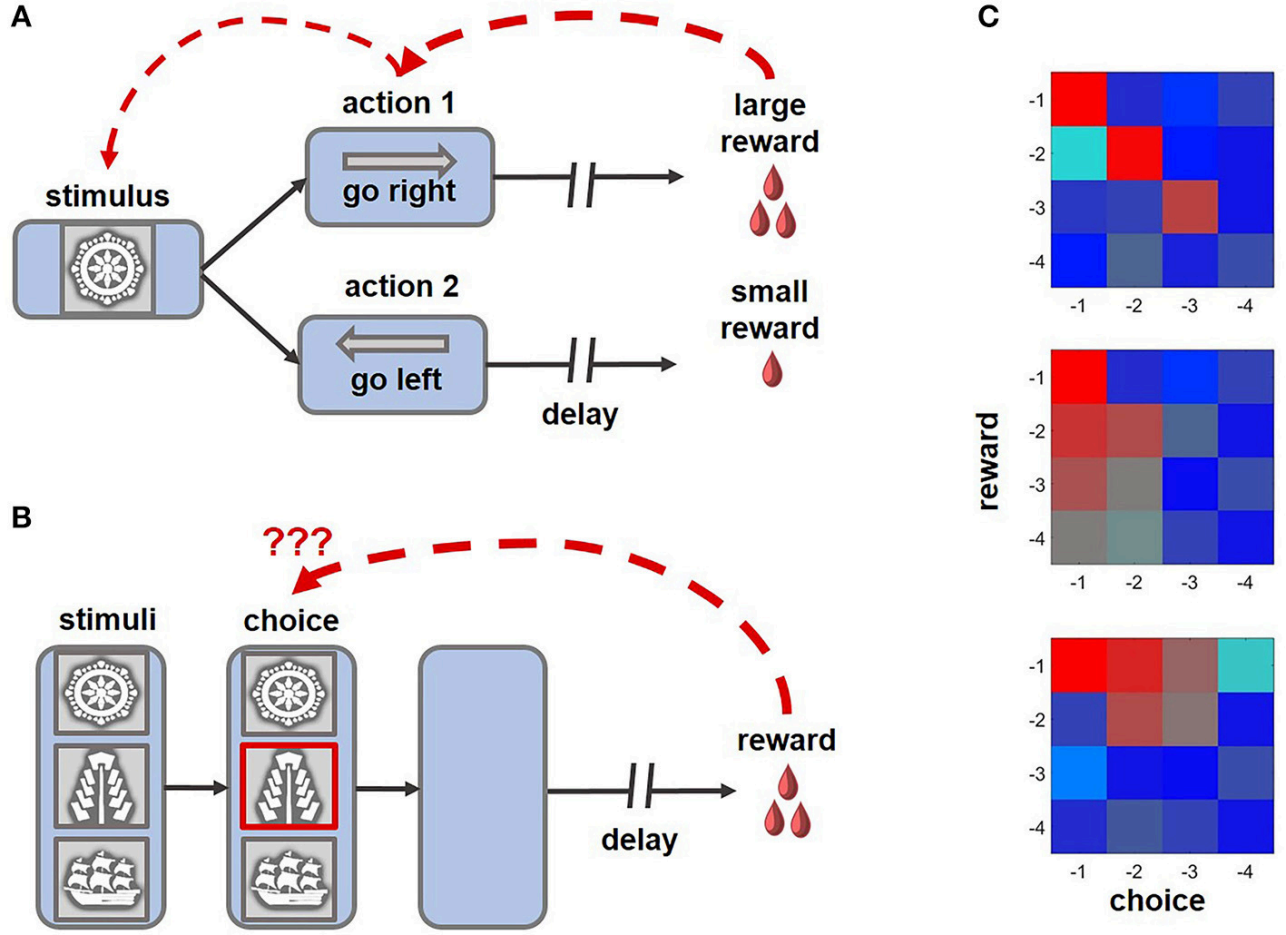
Example tasks highlighting the challenge of credit assignment and learning strategies enabling animals to solve this problem. **(A)** An example of a distal reward task that can be successfully learned with eligibility traces and TD rules, where intermediate choices can acquire motivational significance and subsequently reinforce preceding decisions (ex., Pasupathy and Miller, 2005; Histed et al., 2009). **(B)** In this version of the task, multiple cues are present at the time of choice, only one of which is meaningful for obtaining rewards. After a brief presentation, the stimuli disappear, requiring an animal to solve a complex structural and temporal credit assignment problem (ex., Noonan et al., 2010, 2017; Niv et al., 2015; Asaad et al., 2017; while the schematic of the task captures the challenge of credit assignment, note that in some experimental variants of the behavioral paradigm stimuli disappeared before an animal revealed its choice, whereas in others the cues remained on the screen until the trial outcome was revealed). Under such conditions, learning based on eligibility traces is suboptimal, as non-specific reward signals can reinforce visual cues that did not meaningfully contribute, but occurred close, to beneficial outcomes of behavior. **(C)** On reward tasks, similar to the one shown in **(B)**, the impact of previous decisions and associated rewards on current behavior can be assessed by performing regression analyses (Jocham et al., 2016; Noonan et al., 2017). Here, the color of each cell in a matrix represents the magnitude of the effect of short-term choice and outcome histories, up to 4 trials into the past (red-strong influence; blue-weak influence on the current decision). Top: an animal learning based on the causal relationship between outcomes and choices (i.e., contingent learning). Middle: each choice is reinforced by a combined history of rewards (i.e., decisions are repeated if beneficial outcomes occur frequently). Bottom: the influence of recent rewards spreads to unrelated choices.

When outcomes follow choices after short delays (Figure 1A), the credit for distal rewards can frequently be assigned by establishing an eligibility trace, a sustained memory of the recent activity that renders synaptic connections malleable to modification over several seconds. Eligibility traces can persist as elevated levels of calcium in dendritic spines of post-synaptic neurons (Kötter and Wickens, 1995) or as a sustained neuronal activity throughout the delay period (Curtis and Lee, 2010) to allow for synaptic changes in response to reward signals. Furthermore, spike-timing dependent plasticity can be influenced by neuromodulator input (Izhikevich, 2007; Abraham, 2008; Fisher et al., 2017). For example, the magnitude of short-term plasticity can be modulated by DA, acetylcholine and noradrenaline, which may even revert the sign of the synaptic change (Matsuda et al., 2006; Izhikevich, 2007; Seol et al., 2007; Abraham, 2008; Zhang et al., 2009). Sustained neural activity has been observed in the PFC and striatum (Jog et al., 1999; Pasupathy and Miller, 2005; Histed et al., 2009; Kim et al., 2009, 2013; Seo et al., 2012; Her et al., 2016), as well as the sensory cortices after experience with consistent pairings between the stimuli and outcomes separated by predictable delays (Shuler and Bear, 2006).

On extended timescales, when multiple actions need to be performed sequentially to reach a final goal, the distal reward problem can be solved by assigning motivational significance to intermediate choices that can subsequently reinforce preceding decisions, such as in TD learning models (Montague et al., 1996; Sutton and Barto, 1998; Barto, 2007). Assigning values to these intervening steps according to expected future rewards allows to break complex temporal credit assignment problems into smaller and easier tasks. There is ample evidence for TD learning in humans and other animals that on the neural level is supported by transfer of DA responses from the time of reward delivery to preceding cues and actions (Montague et al., 1996; Schultz, 1998a,b; Walsh and Anderson, 2014).

Both TD learning and eligibility traces offer elegant solutions to the distal reward problem, and models based on cooperation between these two mechanisms can predict animal behavior as well as neuronal responses to rewards and predictive stimuli (Pan et al., 2005; Bogacz et al., 2007). Yet assigning credit based on eligibility traces can be suboptimal when an animal interacts with many irrelevant stimuli prior to or during the delay (Figure 1B). Under such conditions sensory areas remain responsive to distracting stimuli and the arrival of non-specific reward signals can reinforce intervening cues that did not meaningfully contribute, but occurred close, to the outcome of behavior (FitzGerald et al., 2013; Xu, 2017).

## The role of the PFC in structural credit assignment

Several recent studies have investigated the neural mechanisms of appropriate credit assignment in challenging tasks where only a few of the multitude of cues predict rewards reliably. Collectively, this work has provided compelling support for causal contributions of the PFC to structural credit assignment. For example, Asaad et al. (2017) examined the activity of neurons in monkey dlPFC while subjects were performing a delayed learning task. The arrangement of the stimuli varied randomly between trials and within each block either the spatial location or stimulus identity was relevant for solving the task. The monkeys' goal was to learn by trial-and-error to select one of the four options that led to rewards according to current rules. When stimulus identity was relevant for solving the task, neural activity in the dlPFC at the time of feedback reflected both the relevant cue (regardless of its spatial location) and the trial outcome, thus integrating the information necessary for credit assignment. Such responses were strategy-selective: these neurons did not encode cue identity at the time of feedback when it was not necessary for learning in the spatial location task, in which making a saccade to the same position on the screen was reinforced within a block of trials. Previous research has similarly indicated that neurons in the dlPFC respond selectively to behaviorally-relevant and attended stimuli (Lebedev et al., 2004; Markowitz et al., 2015) and integrate information about prediction errors, choice values as well as outcome uncertainty prior to trial feedback (Khamassi et al., 2015).

The activity within the dlPFC has been linked to structural credit assignment through selective attention and representational learning (Niv et al., 2015). Under conditions of reward uncertainty and unknown relevant task features, human participants opt for computational efficiency and engage in a serial-hypothesis-testing strategy (Wilson and Niv, 2011), selecting one cue and its anticipated outcome as the main focus of their behavior, and updating the expectations associated exclusively with that choice upon feedback receipt (Akaishi et al., 2016). Niv and colleagues tested participant on a three-armed bandit task, where relevant stimulus dimensions (i.e., shape, color or texture) predicting outcome probabilities changed between block of trials (Niv et al., 2015). In such multidimensional environment, reinforcement-driven learning was aided by attentional control mechanisms that engaged the dlPFC, intraparietal cortex, and precuneus.

In many tasks, the credit for outcomes can be assigned according to different rules: based on the causal relationship between rewards and choices (i.e., contingent learning), their temporal proximity (i.e., when the reward is received shortly after a response), or their statistical relationship (when an action has been executed frequently before beneficial outcomes; Jocham et al., 2016; Figure 1C). The analyses presented in papers discussed above did not allow for the dissociation between these alternative strategies of credit assignment. By testing human participants on a task with continuous stimulus presentation, instead of a typical trial-by-trial structure, Jocham et al. (2016) demonstrated that the tendency to repeat choices that were immediately followed by rewards and causal learning operate in parallel. In this experiment, activity within another subregion of the PFC, the OFC, was associated with contingent learning. Complementary work in monkeys revealed that the OFC contributes causally to credit assignment (Noonan et al., 2010): animals with OFC lesions were unable to associate a reward with the choice on which it was contingent and instead relied on temporal and statistical learning rules. In another recent paper, Noonan and colleagues (2017) extended these observations to humans, demonstrating causal contributions of the OFC to credit assignment across species. The participants were tested on a three-choice probabilistic learning task. The three options were presented simultaneously and maintained on the screen until the outcome of a decision was revealed, thus requiring participants to ignore irrelevant distractors. Notably, only patients with lateral OFC lesions displayed any difficulty in learning the task, whereas damage to the medial OFC or dorsomedial PFC preserved contingent learning mechanisms. However, it is presently unknown whether lesions to the dlPFC or ACC affect such causal learning.

In another test of credit assignment in learning, contingency degradation, the subjects are required to track causal relationships between the stimuli or actions and rewards. During contingency degradation sessions, the animals are still reinforced for responses, but rewards are also available for free. After experiencing non-contingent rewards, control subjects reliably decrease their choices of the stimuli. However, lesions to both the ACC and OFC inhibit contingency degradation (Jackson et al., 2016). Taken together, these observations demonstrate causal contributions of the PFC to appropriate credit assignment in multi-cue environments.

## Cooperation between PFC subregions supports contingent learning in multi-cue tasks

Despite the segregation of temporal and structural aspects of credit assignment in earlier sections of this review, in naturalistic settings the brains frequently need to tackle both problems simultaneously. Here, I overview the evidence favoring a network perspective, suggesting that dynamic cortico-cortical interactions during decision making and outcome valuation enable adaptive solutions to complex spatio-temporal credit assignment problems. It has been previously suggested that feedback projections from cortical areas occupying higher levels of processing hierarchy, including the PFC, can aid in attribution of outcomes to individual decisions by implementing attention-gated reinforcement learning (Roelfsema and van Ooyen, 2005). Similarly, recent theoretical work has shown that even complex multi-cue and multi-step problems can be solved by an extended cascade model of synaptic memory traces, in which the plasticity is modulated not only by the activity within a population of neurons, but also by feedback about executed decisions and resulting rewards (Urbanczik and Senn, 2009; Friedrich et al., 2010, 2011). Contingent learning, according to these models, can be supported by the communication between neurons encoding available options, committed choices and outcomes of behavior during decision making and feedback monitoring. For example, at the time of outcome valuation, information about recent choices can be maintained as a memory trace in the neuronal population involved in action selection or conveyed by an efference copy from an interconnected brain region (Curtis and Lee, 2010; Khamassi et al., 2011, 2015). Similarly, reinforcement feedback is likely communicated as a global reward signal (ex., DA release) as well as projections from neural populations engaged in performance monitoring, such as those within the ACC (Friedrich et al., 2010; Khamassi et al., 2011). The complexity of reciprocal and recurrent projections spanning the PFC (Barbas and Pandya, 1989; Felleman and Van Essen, 1991; Elston, 2000) may enable this network to implement such learning rules, integrating the information about the task, executed decisions and performance feedback.

In many everyday decisions, the options are compared across multiple features simultaneously (ex., by considering current context, needs, available reward types, as well as delay and effort costs). Neurons in different subregions of the PFC exhibit rich response properties, signaling these features of the task at various time epochs within a trial. For example, reward selectivity in response to predictive stimuli emerges earlier in the OFC and may then be passed to the dlPFC that encodes both the expected outcome and the upcoming choice (Wallis and Miller, 2003). Similarly, on trials where options are compared based on delays to rewards, choices are dependent on interactions between the OFC and dlPFC (Hunt et al., 2015). Conversely, when effort costs are more meaningful for decisions, it is the ACC that influences choice-related activity in the dlPFC (Hunt et al., 2015). The OFC is required not only for the evaluation of stimuli, but also more complex abstract rules, based on rewards they predict (Buckley et al., 2009). While both the OFC and dlPFC encode abstract strategies (ex., persisting with recent choices or shifting to a new response), such signals appear earlier in the OFC and may be subsequently conveyed to the dlPFC where they are combined with upcoming response (i.e., left vs. right saccade) encoding (Tsujimoto et al., 2011). Therefore, the OFC may be the first PFC subregion to encode task rules and/or potential rewards predicted by sensory cues; via cortico-cortical projections, this information may be subsequently communicated to the dlPFC or ACC (Kennerley et al., 2009; Hayden and Platt, 2010) to drive strategy-sensitive response planning.

The behavioral strategy that the animal follows is influenced by recent reward history (Cohen et al., 2007; Pearson et al., 2009). If its choices are reinforced frequently, the animal will make similar decisions in the future (i.e., exploit its current knowledge). Conversely, unexpected omission of expected rewards can signal a need for novel behaviors (i.e., exploration). Neurons in the dlPFC carry representations of planned as well as previous choices, anticipate outcomes, and jointly encode the current decisions and their consequences following feedback (Seo and Lee, 2007; Seo et al., 2007; Tsujimoto et al., 2009; Asaad et al., 2017). Similarly, the ACC tracks trial-by-trial outcomes of decisions (Procyk et al., 2000; Shidara and Richmond, 2002; Amiez et al., 2006; Quilodran et al., 2008) as well as reward and choice history (Seo and Lee, 2007; Kennerley et al., 2009, 2011; Sul et al., 2010; Kawai et al., 2015) and signals errors in outcome prediction (Kennerley et al., 2009, 2011; Hayden et al., 2011; Monosov, 2017). At the time of feedback, neurons in the OFC encode committed choices, their values and contingent rewards (Tsujimoto et al., 2009; Sul et al., 2010). Notably, while the OFC encodes the identity of expected outcomes and the value of the chosen option after the alternatives are presented to an animal, it does not appear to encode upcoming decisions (Tremblay and Schultz, 1999; Wallis and Miller, 2003; Padoa-Schioppa and Assad, 2006; Sul et al., 2010; McDannald et al., 2014), therefore it might be that feedback projections from the dlPFC or ACC are required for such activity to emerge at the time of reward feedback.

To capture the interactions between PFC subregions in reinforcement-driven learning, Khamassi and colleagues have formulated a computation model in which action values are stored and updated in the ACC and then communicated to the dlPFC that decides which action to trigger (Khamassi et al., 2011, 2013). This model relies on meta-learning principles (Doya, 2002), flexibly adjusting the exploration-exploitation parameter based on performance history and variability in the environment that are monitored by the ACC. The explore-exploit parameter then influences action-selection mechanisms in the dlPFC, prioritizing choice repetition once the rewarded actions are discovered and encouraging switching between different options when environmental conditions change. In addition to highlighting the dynamic interactions between the dlPFC and ACC in learning, the model similarly offers an elegant solution to the credit assignment problem by restricting value updating only to those actions that were selected on a given trial. This is implemented by requiring the prediction error signals in the ACC to coincide with a motor efference copy sent by the premotor cortex. The model is endorsed with an ability to learn meta-values of novel objects in the environment based on the changes in the average reward that follow the presentation of such stimuli. While the authors proposed that such meta-value learning is implemented by the ACC, it is plausible that the OFC also plays a role in this process based on its contributions to stimulus-outcome and state learning (Wilson et al., 2014; Zsuga et al., 2016). Intriguingly, this model could reproduce monkey behavior and neural responses on two tasks: four-choice deterministic and two-choice probabilistic paradigms, entailing a complex spatio-temporal credit assignment problem as the stimuli disappeared from the screen prior to action execution and outcome presentation (Khamassi et al., 2011, 2013, 2015). Model-based analyses of neuronal responses further revealed that information about prediction errors, action values and outcome uncertainty is integrated both in the dlPFC and ACC, but at different timepoints: before trial feedback in the dlPFC and after feedback in the ACC (Khamassi et al., 2015).

**Figure 2:**
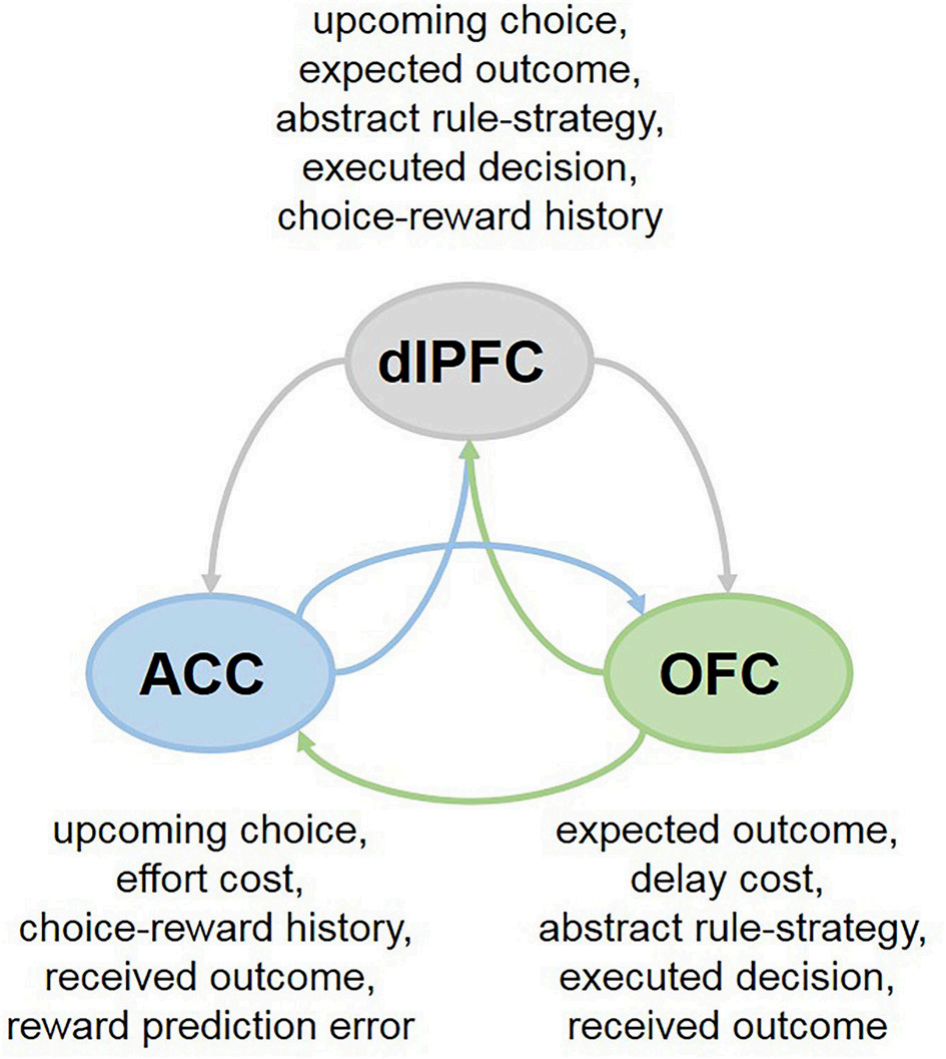
Cooperation between PFC subregions in multi-cue tasks. In many everyday decisions, the options are compared across multiple features simultaneously (ex., by considering current context, needs, available reward types, as well as delay and effort costs). Neurons in different subregions of the PFC exhibit rich response properties, integrating many aspects of the task at hand. The OFC, ACC and dlPFC communicate extensively, sharing the information about presented options, executed decisions and received rewards, which can enable them to assign credit for outcomes to choices on which they are contingent.

Collectively, these findings highlight the heterogeneity of responses in each PFC subregion that differ in temporal dynamics within a single trial and suggest that the cooperation between the OFC, ACC and dlPFC may support flexible, strategy- and context-dependent choices. This network perspective further suggests that individual PFC subregions may be less specialized in their functions than previously thought. For example, in primates both the ACC and dlPFC participate in decisions based on action values (Hunt et al., 2015; Khamassi et al., 2015). And more recently, it has been demonstrated that the OFC is involved in updating action-outcome values as well (Fiuzat et al., 2017). Analogously, while it has been proposed that the OFC is specialized for stimulus-outcome and ACC for action-outcome learning (Rudebeck et al., 2008), lesions to the ACC have been similarly reported to impair stimulus-based reversal learning (Chudasama et al., 2013), supporting shared contributions of the PFC subregions to adaptive behavior. Indeed, these brain regions communicate extensively, sharing the information about presented options, executed decisions and received rewards (Figure 2), which can enable them to assign credit for outcomes to choices on which they are contingent (Urbanczik and Senn, 2009; Friedrich et al., 2010, 2011). Attention-gated learning likely relies on the cooperation between PFC subregions as well: for example, coordinated and synchronized activity between the ACC and dlPFC aids in goal-directed attentional shifting and prioritization of task-relevant information (Womelsdorf et al., 2014; Oemisch et al., 2015; Voloh et al., 2015).

Functional connectivity within the PFC can support contingent learning on shorter timescales (ex., across trials within the same task), when complex rules or stimulus-action-outcome mappings are switching frequently (Duff et al., 2011; Johnson et al., 2016). Under such conditions, the same stimuli can carry different meaning depending on task context or due to changes in the environment (ex., serial discrimination-reversal problems) and the PFC neurons with heterogeneous response properties may be better targets for modification, allowing the brain to exert flexible, rapid and context-sensitive control over behavior (Asaad et al., 1998; Mansouri et al., 2006). Indeed, it has been shown that rule and reversal learning induce plasticity in OFC synapses onto the dorsomedial PFC (encompassing the ACC) in rats (Johnson et al., 2016). When motivational significance of reward-predicting cues fluctuates frequently, neuronal responses and synaptic connections within the PFC tend to update more rapidly (i.e., across block of trials) compared to subcortical structures and other cortical regions (Padoa-Schioppa and Assad, 2008; Morrison et al., 2011; Xie and Padoa-Schioppa, 2016; Fernández-Lamo et al., 2017; Saez et al., 2017). Similarly, neurons in the PFC promptly adapt their responses to incoming information based on the recent history of inputs (Freedman et al., 2001; Meyers et al., 2012; Stokes et al., 2013). Critically, changes in the PFC activity closely track behavioral performance (Mulder et al., 2003; Durstewitz et al., 2010), and interfering with neural plasticity within this brain area prevents normal responses to contingency degradation (Swanson et al., 2015).

When the circumstances are stable overall and the same cues or actions remain reliable predictors of rewards, long-range connections between the PFC, association and sensory areas can support contingent learning on prolonged timescales. Neurons in the lateral intraparietal area demonstrate larger post-decisional responses and enhanced learning following choices that predict final outcomes of sequential behavior in a multi-step and -cue task (Gersch et al., 2014). Such changes in neuronal activity likely rely on information about task rules conveyed by the PFC directly or via interactions with neuromodulatory systems. These hypotheses could be tested in future work.

In summary, dynamic interactions between subregions of the PFC can support contingent learning in multi-cue environments. Furthermore, via feedback projections, the PFC can guide plasticity in other cortical areas associated with sensory and motor processing (Cohen et al., 2011). This account suggests that lesion experiments targeting a localized PFC subregion will be insufficient to gain fine-grained understanding of credit assignment during learning and instead poses refined questions for future research, shifting the focus from focal manipulations to experimental techniques targeting cortico-cortical projections. To gain novel insights into functional connectivity between PFC subregions, it will be critical to assess neural correlates of contingent learning in the OFC, ACC, and dlPFC simultaneously in the context of the same task. In humans, functional connectivity can be assessed by utilizing coherence, phase synchronization, Granger causality and Bayes network approaches (Bastos and Schoffelen, 2016; Mill et al., 2017). Indeed, previous studies have linked individual differences in cortico-striatal functional connectivity to reinforcement-driven learning (Horga et al., 2015; Kaiser et al., 2017) and future work could focus on examining cortico-cortical interactions in similar paradigms. To probe causal contributions of projections spanning the PFC, future research may benefit from designing multi-cue tasks for rodents and taking advantage of recently developed techniques (i.e., chemo- and opto-genetic targeting of projection neurons followed by silencing of axonal terminals to achieve pathway-specific inhibition; Deisseroth, 2010; Sternson and Roth, 2014) that afford increasingly precise manipulations of cortico-cortical connectivity. It should be noted, however, that most experiments to date have probed the contributions of the PFC to credit assignment in primates, and functional specialization across different subregions may be even less pronounced in mice and rats. Finally, as highlighted throughout this review, the recent progress in understanding the neural mechanisms of credit assignment has relied on introduction of more complex tasks, including multi-cue and probabilistic choice paradigms. While such tasks better mimic the naturalistic problems that the brains have evolved to solve, they also produce behavioral patterns that are more difficult to analyze and interpret (Scholl and Klein-Flügge, 2017). As such, computational modeling of the behavior and neuronal activity may prove especially useful in future work on credit assignment.

## Author contributions

The author confirms being the sole contributor of this work and approved it for publication.

### Conflict of interest statement

The author declares that the research was conducted in the absence of any commercial or financial relationships that could be construed as a potential conflict of interest.
